# Dietary salt levels affect digestibility, intestinal gene expression, and the microbiome, in Nile tilapia (*Oreochromis niloticus*)

**DOI:** 10.1371/journal.pone.0202351

**Published:** 2018-08-23

**Authors:** Eyal Hallali, Fotini Kokou, Tapan Kumar Chourasia, Tali Nitzan, Pazit Con, Sheenan Harpaz, Itzhak Mizrahi, Avner Cnaani

**Affiliations:** 1 Institute of Animal Science, Agricultural Research Organization, Rishon LeZion, Israel; 2 Faculty of Life Sciences, Bar-Ilan University, Ramat-Gan, Israel; 3 Department of Life Sciences, Ben-Gurion University of the Negev, Beer-Sheva, Israel; University of Hyderabad, INDIA

## Abstract

Nile tilapia (*Oreochromis niloticus*) is the world’s most widely cultured fish species. Therefore, its nutritional physiology is of great interest from an aquaculture perspective. Studies conducted on several fish species, including tilapia, demonstrated the beneficial effects of dietary salt supplementation on growth; however, the mechanism behind these beneficial effects is still not fully understood. The fish intestine is a complex system, with functions, such as nutrient absorption, ion equilibrium and acid-base balance that are tightly linked and dependent on each other's activities and products. Ions are the driving force in the absorption of feed components through pumps, transporters and protein channels. In this study, we examined the impact of 5% increase in dietary NaCl on protein, lipid, ash and dry matter digestibility, as well as on the expression of intestinal peptide transporters (PepTs) and ion pumps (Na^+^/K^+^-ATPase, V-H^+^-ATPase, N^+^/H^+^-Exchanger) in Nile tilapia. In addition, effects on the gut microbiome were evaluated. Our results show that dietary salt supplementation significantly increased digestibility of all measured nutritional components, peptide transporters expression and ion pumps activity. Moreover, changes in the gut microbial diversity were observed, and were associated with lipid digestibility and Na^+^/K^+^-ATPase expression.

## Introduction

The fish intestine is a complex system with many functions. In addition to its obvious role in feed digestion and nutrient absorption, the intestine has a central role in maintaining ion equilibrium, acid-base balance and carbon dioxide emissions [1]. Furthermore, these functions are tightly linked and dependent on each other's activities and products. Ions are the driving force in the absorption of feed components through pumps, transporters and protein channels [2,3]. Expression and activity of these proteins are under complex regulation, resulting from the different roles of the intestine. The activity of numerous transporters involved in nutrient absorption within the intestine is dependent on pH, metabolites concentration and electric potential [4–7]. The surrounding water, feed composition, microbial activity and the endogenic metabolic products, influence these environmental conditions.

Several studies have shown that the addition of salt to the fish feed has a significant impact on growth, feed utilization and physiological parameters in the intestine and blood [8–13]. Studies aimed at understanding the specific effects caused by dietary salt supplementation, found effects on both the basic intestinal enzymatic activity [12], and on the intestinal bacterial populations [14]. Sodium has an important role in the intestinal absorption of numerous dietary nutrients, mediated by several transporters which have Na^+^-dependent activity [2,15,16].

Protein digestion begins in the stomach and continues in the intestine where it is broken down into free amino acid (FAA) or small peptides (di- and tri-peptides) by several proteases. Small peptides absorption is currently known to be mediated by one type of protein system in the intestinal brush borders, the Peptide Transporter (PepT). This solute carrier transports di- and tri-peptides into the enterocytes and can be found in a wide range of organisms [17], including fish [18]. PepT1, which in fish has two paralogs, PepT1a and PepT1b (coded by *slc15a1a* and *slc15a1b*, respectively), is known as a high capacity and low affinity transporter, while PepT2 (coded by *slc15a2*) is known as a high affinity and low capacity transporter [19,20]. The absorption of small peptides interceded by PepT, is coupled with proton absorption [16]. NHE3 and V-H^+^-ATPase are two transporters that excrete protons from the cells and are relatively abundant in the intestine. Both proteins were studied in fish for their role in ion and pH regulation [21,22] but not for their relationships with peptide transporters. Based on mammalian models, NHE3 was considered as the facilitator of the proton gradient that enables PepT activity [3]. Evidence of the relationship between these proteins in fish was provided by Con et al. [4], who found a significant positive correlation between the gene expressions of V-H^+^-ATPase and PepTs in the intestine of Mozambique tilapia (*Oreochromis mossambicus*). Little is known about the effect of dietary Na^+^ on intestinal nutrient absorption and on the relationships between the expression of PepTs and apparent digestibility coefficient (ADC) of proteins. Considering the effect of luminal sodium ions on NHE3 and V-H^+^-ATPase activities, and the correlation between the expression of these transporters and of di/tri peptide transporters, we hypothesized that the addition of sodium to fish feed will enhance the uptake of short peptides, resulting in higher protein digestibility.

Nile tilapia (*Oreochromis niloticus*), a tilapiine species of the family Cichlidae, is one of the world’s most widely cultured fish species, partially due to its ability to utilize a wide range of nutritional sources and suitability to various culture systems. Since PepT systems were found to participate in protein utilization and their activity is dependent on ions and the pH of the lumen, they are prime candidates for nutritional research [23]. The purpose of the present study was to examine the impact of dietary NaCl levels on nutrient absorption and PepTs expression in Nile tilapia.

## Methods

### Animals

The fish used in this study were from the Chitralada strain of *O*. *niloticus* (8 months old, 125±15 g), obtained from the Dor Aquaculture Research Station, Israel. As tilapia’s aquaculture is based on all-male populations and to avoid sex-based variation, only male fish were used in this study.

### Ethics

This study was approved by the Agricultural Research Organization Committee for Ethics in Using Experimental Animals, and was carried out in compliance with the current laws governing biological research in Israel (Approval number: IL-620/15).

### Feces collection system

Two feces-collecting systems were set-up according to the one described by Lee [24]. Each system included six conical shaped tanks (70 liter each), with a feces-trap at the bottom of each tank. Every system of six tanks was fully recirculated, connected to a common plastic bead biofilter with submersible heater and air supply. The system run with freshwater and flow velocity was adjusted to maximize collection of the feces in the trap and was set at 20 l h^-1^. A Styrofoam box filled with ice was inserted below each trap, to reduce microbial degradation during the collection period.

### Diet preparation

The diet used in this study was in the form of 3 mm floating extruded pellets (1103F0, Raanan Feeds, Israel). 5% salt-enriched diet (SED) was prepared by homogeneously spraying NaCl solution on the commercial diet (CD), followed by drying at 60°C for 12 hours. This resulted in a slight proportional reduction in the ration of all other ingredients. The percentage of feed nutrients was measured by chemical composition analyses, as described below in the analytical methods section (Table 1).

**Table 1:** Chemical composition of the commercial diet and the salt-enriched diet (%).

	Commercial diet (CD)*	Salt-enriched diet (SED)*
Protein	30.43±0.88	27.25±1.76
Fat	3.68±0.52	3.29±0.07
Salt	3.05±0.06	7.97±0.05
Ash	8.32±0.04	12.89±0.18
Moisture	11±0.01	12.6±0.19

*Values are expressed as mean ± SD.

### Experimental setup and samples collection

Twelve fish were randomly distributed into the conical shaped tanks, one fish per tank, with 12:12 h light-dark photoperiod, and water temperature was kept at 25±1°C. Levels of dissolved oxygen and pH were 6.54±0.8 mg l^-1^ and 7.73±0.46, respectively, throughout the experimental period. Ammonia and nitrite were measured once a week and were at non-detectable levels. The fish were acclimatized to the system for 5 days followed by a trial period of 42 days. The experiment consisted of two treatments, six tanks in which the fish were fed with SED and six tanks in which the fish were fed with CD. Fish were fed to satiation twice a day, at 8:00 AM and 5:00 PM. Daily feed consumption was recorded for each fish. Prior to feeding, feces were collected from the trap, transferred to 50 ml tube and centrifuged at 4,400*g* for 10 min at 4°C, after which the supernatant was discarded. The feces were then stored at -20°C until analyzed. Fecal samples from each tank were pooled at the end of experiment. Specific growth rate (SGR) was calculated according to the following equation: SGR (% day^−1^) = 100 × (*ln* final body weight–*ln* initial body weight) / days of growth. The feed conversion ratio (FCR) was calculated as given food / weight gain. At the end of the trial, all fish were sacrificed and sampled. The fish intestine was removed, cleaned from remaining connective tissues and fat, and divided into three separate sections: anterior intestine (AI), middle intestine (MI) and posterior intestine (PI). Tissue samples were kept in RNA*later* buffer (Ambion, AM7021) at -20°C for gene expression analysis and in SEI buffer (250 mmol l^-1^ sucrose, 10 mmol l^-1^ Na_2_EDTA, 50 mmol l^-1^ imidazole and adjusted to pH 7.4) at -80°C for ATPase activity assay. In addition, anterior and posterior gut tissue samples were kept in sterile tubes at -80°C for gut microbiome analysis.

### Analytical methods

Feed and feces chemical composition followed the methods described by Jimoh et al. and Nengas et al. [25,26]. Prior to analyses, frozen feed and feces were dried in an oven at 105°C for 24 hours, and ground to a powder. Crude protein (%N×6.25) was determined by the Kjeldahl method using an Auto Kjeldahl System (Foss, Kjeltec 8400). Feed moisture was determined as a percentage of weight loss after drying a known weight of the sample at 105°C for 24 h. Ash was determined by a muffler furnace at 400°C for 9 h. Crude lipid was determined by Sulfo-Phospho-Vanillin Reaction, with olive oil as a standard. Acid Insoluble Ash (AIA) was determined utilizing a muffler furnace at 550°C for 12 h after the sample was digested with boiled 7% HCl for 5 min [27]. All chemical analyses for each fish were carried out in triplicate. Calculations of apparent digestibility coefficients (ADC) for protein, lipid, ash and dry matter were determined using the following equation [25]:

$$ADC\;of\;nutrients\;(\%)=100*\left(1-(\frac{\%dietary\;AIA}{\%fecal\;AIA})*(\frac{\%fecal\;nutrient}{\%dietary\;nutrient})\right)$$

### Relative gene expression

RNA extraction, cDNA synthesis and quantitative real-time PCR (qPCR) analyses were performed as described in our previous studies on the tilapia intestine, [4,28] with the same primers sets (listed in Table 2). Briefly, total RNA was extracted using Trizol reagent, purified from DNA contamination using TURBO DNA-free Kit (Ambion), quantified with Nano-Drop spectrophotometer (Thermo Scientific), and then reverse- transcribed into cDNA using the Verso cDNA Synthesis Kit (Thermo Scientific). qPCR reactions were conducted using ABsoluteTM Blue QPCR SYBR Green ROX Mix in a 10ul reaction volume on an Eco Real-Time PCR System (Illumina) as follows: 95°C for 15 min, followed by 40 cycles of 95°C for 15 s, 60°C for 30 s, and 72°C for 30 s. Relative quantification to the reference gene EF1α was calculated using the ΔCt method [29].

**Table 2:** Sequences of primers used for qPCR.

Gene	GeneBank	Forward	Reverse
***slc15a1a***	XM_003459630	taaaaccctgcctgacttcc	aatcctcattagccccaaaa
***slc15a1b***	XM_003447363	ccaagccagaacaaggtaaca	ggctcaattagtcccaagtcc
***slc15a2***	XM_003454878	ctgcgaacgcttctcctact	cgctgaaagcatggtagaca
***ef-1a***	XM_003458541	tcaacgctcaggtcatcatc	acggtcgatcttctcaacca
***atp6v1a***	AB369668	ccacagctcagagcgacaaca	gcatactcggccttgatcttg
***slc9a3***	AB326212.1	aagcggcacccatcactaca	gagccagcaaaccagaatcca
***atp1a3***	AF109409.1	atgaagcgtcagcctaggaa	tcccagagcctggatcatac

### ATPase assay

An ATPase assay [30,31], was adapted to determine both the ouabain (Na^+^/K^+^-ATPase inhibitor) and bafilomycin (V-H^+^-ATPase inhibitor)-sensitive ATPase activities [28]. All three intestinal sections (Anterior, Mid and Posterior) were stored in SEI buffer at -80°C until assays were performed. Tissues were thawed and homogenized with the addition of 0.5% sodium deoxycholic acid on ice and immediately centrifuged at 5,000 *g* for 30 s to remove insoluble material. Homogenate (10 μl) from each sample was added to nine wells in a 96-well plate. This provided three treatments for each sample [control, ouabain (500 μmol l^–1^) and ouabain (500 μmol l^–1^) + bafilomycin (50 nmol l^–1^)] with triplicate measurements of each treatment. Preliminary experiments demonstrated that maximal inhibition of V-H^+^-ATPase activity was obtained at a concentration range of 10–100 nmol l^–1^. Therefore, 50 nmol l^–1^ was chosen as the appropriate dose for this assay. 150 μl of assay mixture [50 mmol l^–1^ imidazole buffer, 2 mmol l^–1^ phosphoenol pyruvate (PEP), 0.16 mmol l^–1^ NADH, 0.5 mmol l^–1^ ATP, 3.3 U ml^–1^ lactate dehydrogenase (LDH), 3.6 U ml^–1^phosphokinase (PK)] were added to each well, with appropriate drug treatment, and 50 μl of salt solution (50 mmol l^–1^ imidazole, 189 mmol l^–1^ NaCl, 10.5 mmol l^–1^ MgCl_2_, 42 mmol l^–1^ KCl). The microplate was read at a wavelength of 340 nm in a microplate reader (Epoch™, BioTek Instruments, Inc., Winooski, VT, USA) for 20 min. at 15 s intervals. The average rate for each treatment was taken from the stable slope and calculated from a standard curve generated just prior to the assay. Na^+^/K^+^-ATPase activity was obtained by subtracting the ouabain-treated ATPase activity from control ATPase activity. We also modified the assay to assess V-H^+^-ATPase activity by calculating the difference in ATPase activity between the ouabain- and the ouabain + bafilomycin-treated samples.

### Intestinal microbiome analysis

Bacterial DNA was isolated from anterior and posterior intestinal samples using the protocol described by Sun et al. [14]. Excised intestinal tissues were combined in 2.0-ml screw-cap tubes with 0.5-mm and 1-mm silica beads, 400 ml 50 mM Na-phosphate buffer (pH 8.0) and 200 ml of lysis solution containing 5% w/v sodium dodecyl sulfate, 0.5 M Tris-HCl (pH 8.0) and 0.1 M NaCl. Samples were homogenized in a bead-beater for 5 min and centrifuged at 16,000g for 5 min. The supernatant was transferred to new tubes and lysozyme (Sigma, St. Louis, MO) was added to a final concentration of 2 mg/ml followed by incubation at 42°C for 1 h and then at 37°C for 1 h. The solution was sequentially extracted with TE (10 mM Tris-HCl pH 8.0 and 1 mM EDTA), saturated phenol, phenol-chloroform (1:1 v/v), and chloroform-isoamyl alcohol (24:1 v/v). DNA in the aqueous phase was precipitated with 0.1 volume 3 M sodium acetate (pH 5.2) and 0.7 volume isopropanol. DNA concentration was measured using a Nanodrop 2000 UV-Vis Spectrophotometer (Thermo Scientific, Waltham, MA).

PCR-amplified V4 region of 16S rRNA was sequenced using the Illumina MiSeq 2000 Next Generation system. Amplification of the V4 region, using primers 515F and 806R was performed under the following conditions: 94°C for 15 min, followed by 35 cycles of 94°C for 45 s, 50°C for 60 s and 72°C for 90 s, and a final elongation step at 72°C for 10 min. The 380 bp PCR product was cleaned using DNA Clean & Concentrator™ (Zymo Research, Irvine, CA) and quantified for the fragments containing the Illumina adaptors. Amplification involved 15 min at 95°C for initial denaturation and then 40 cycles at 95°C for 10 s followed by annealing at 60°C for 20 s and extension at 72°C for 30 s. Products were quantified using a standard curve with serial DNA concentrations (0.1–10 nM). Samples were equimolarly diluted to a concentration of 0.4 nM and prepared for sequencing according to the manufacturer’s instructions. Data quality control and analyses were performed using the QIIME pipeline [32], following the default settings. Reads were assigned to their designated sample. Then, a length-based filtering)>200 bp) and read-quality filtering were performed. Due to the high variation of sequence depths between samples, they were normalized to the median depth by subsampling (6000 read/sample). Obtained sequences were aligned to define operational taxonomic units (OTUs) for taxonomy assignment. The ‘denovo’ UCLUST method [33] was used to cluster the reads into OTUs at 97% sequence identity. Taxonomy was assigned using the RDP (Ribosomal Database Project) algorithm [34] against the 16S reference database found at: http://qiime.org, designated as ‘most recent Greengenes OTUs’ (13_8 version) [35]. Raw sequences are under the accession number SRP149036 in the Sequence Read Archive (SRA).

The alpha diversity indices Shannon H’, richness, phylogenetic diversity and dominance were calculated using QIIME (*alpha_diversity*.*py*). Correlation analysis of alpha diversity indices with fish growth and physiological parameters were assessed using Spearman (r). The microbial composition was calculated using the QIIME script ‘*beta_diversity*.*py*’. Canonical correspondence analysis (CCA) was performed to arrange microbial species along environmental variables, that were selected based on the correlation analysis. CCA constructs linear combinations of environmental variables, along which the distributions of the microbial species are maximally separated. The ordination diagram visualizes the pattern of community variation and distributions of microbial species along the environmental variables. Taxa with significant differential abundance between the two groups (SED and CD) were detected using QIIME (*differential_abundance*.*py;* DESeq2) [36]. Similarity percentages (SIMPER) analysis was used to determine the genera which contributed most to the discrimination of samples in each group, using Bray Curtis dissimilarity metric.

### Statistical analyses

The effects of dietary salt levels on ADC values, and on gene expression and activity in each intestinal section, were analyzed using t-test, with diets as the independent variant (α = 0.05), and fish as replicates. Correlations between expression levels of the examined genes, and between genes expression and ADC values, were analyzed using Pearson correlation method, with individual fish as replicates (α = 0.05) Data are presented as means ± SEM.

## Results

### Growth and nutrients digestibility

Fish receiving the SED diet had higher growth parameters than the control, with SGR of 1.54±0.1 compare to 1.13±0.2, and daily weight gain of 2.78±0.2 compare to 1.89±0.4. They also had better FCR, 1.78±0.1 compare to 2.02±0.3. However, these differences were not significant.

**Fig 1:**
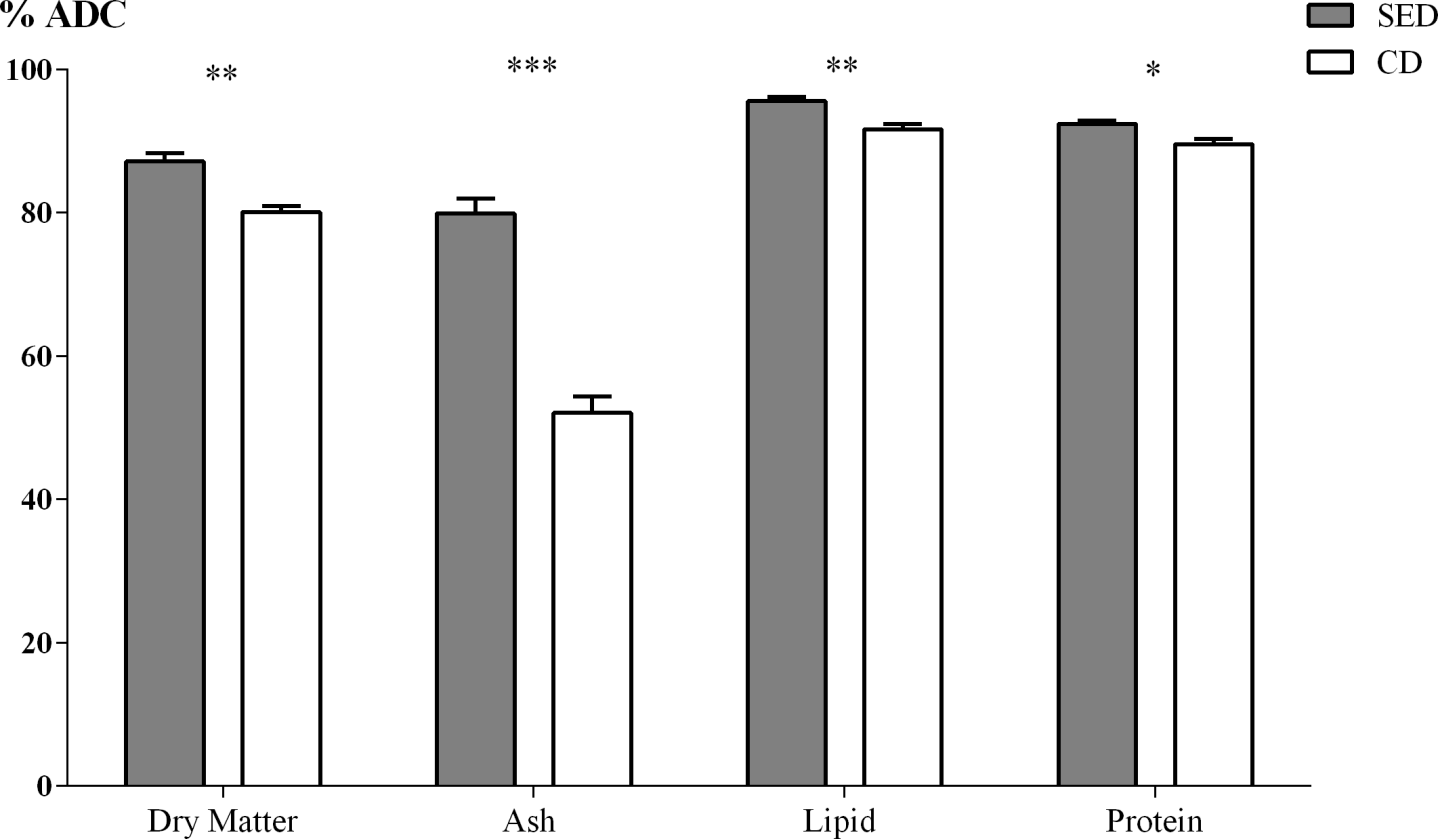
Apparent digestibility coefficient (ADC) values of dry matter, ash, lipid and protein between the treatments (CD = Control Diet; SED = Salt Enriched Diet). N = 6, asterisks indicate a significant difference between the treatments (* P<0.05; ** P<0.01; *** P<0.005).

Fish that were fed CD excreted 9.7% more (dry feces) than the fish that were fed SED: 0.271±0.032g for one gram of body weight compared to 0.247±0.018g for one gram of body weight. Apparent digestibility coefficient (ADC) for Nile tilapia of dry matter, ash, lipid and protein were significantly affected by the dietary treatment that had salt supplementation (Fig 1). The ADC of dry matter, ash, lipid and protein of the fish that were fed SED were significantly higher than the fish that were fed CD (P = 0.001, P<0.0001, P = 0.002 and P = 0.011, respectively).

### Peptide transporters

**Fig 2:**
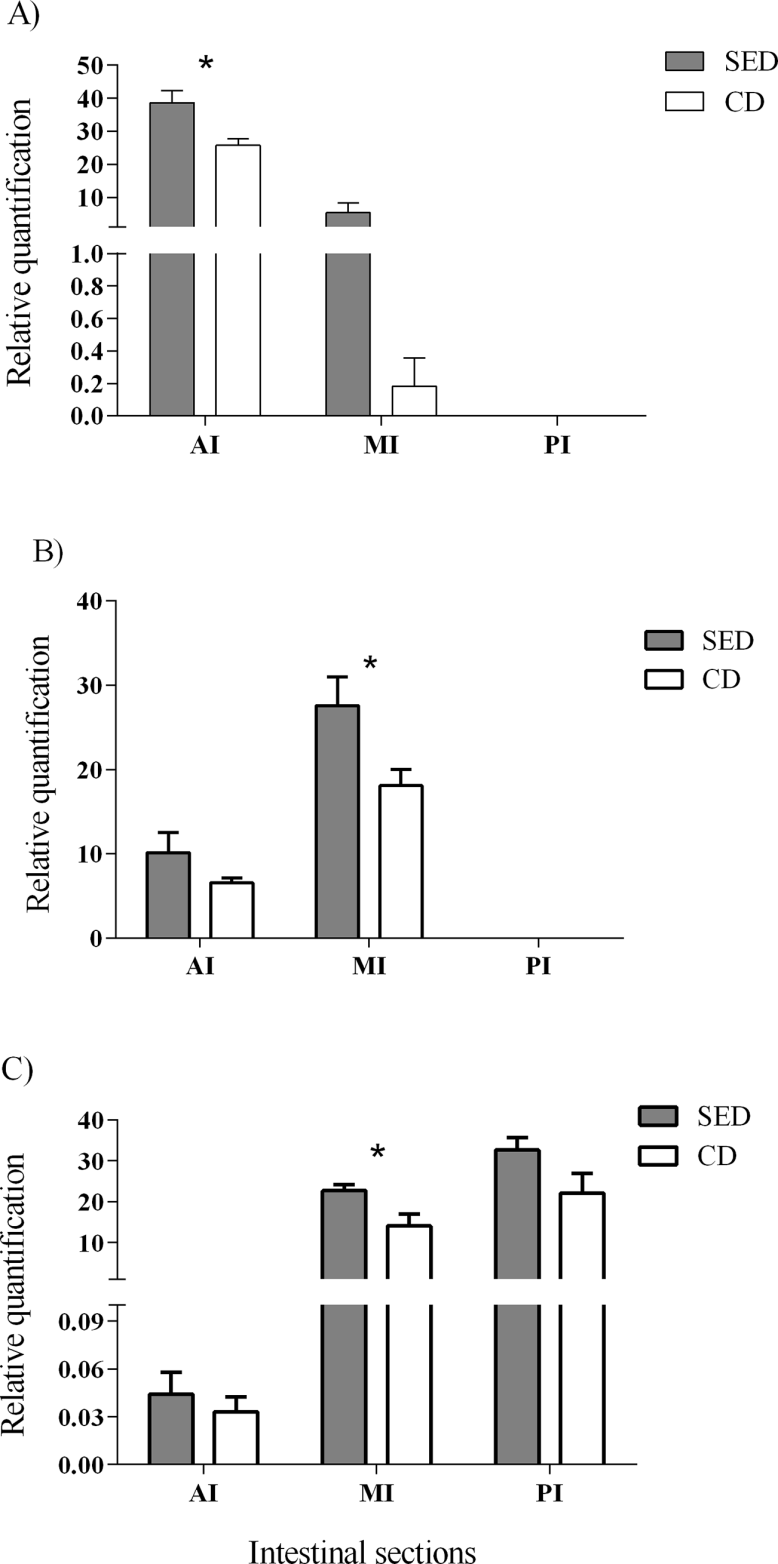
Relative quantification of mRNA expression of *slc15a1a* (A), *slc15a1b* (B) and *slc15a2* (C) at the anterior (AI), middle (MI) and posterior (PI) intestine between the treatments. N = 6, asterisks indicate a significant difference between the treatments (P<0.05).

**Fig 3:**
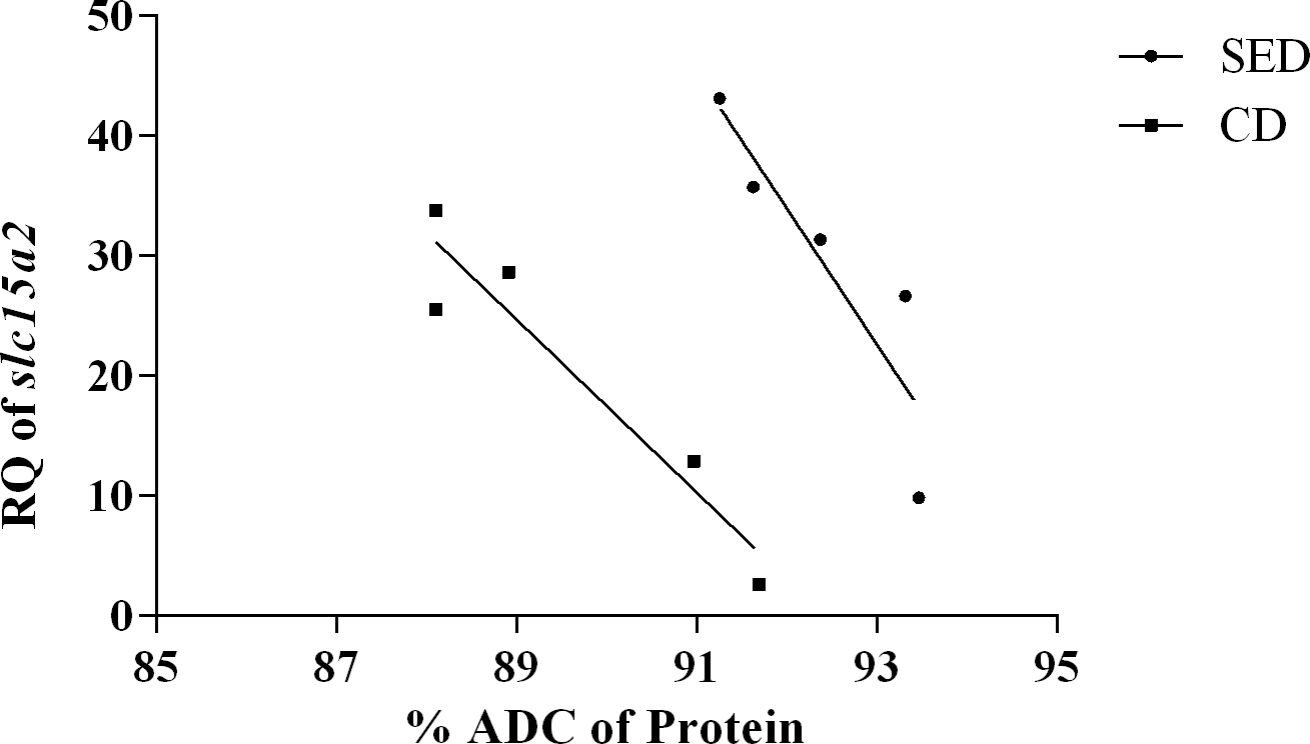
Correlation between the relative quantification (RQ) of mRNA expression of *slc15a2* at the posterior intestine and the ADC of protein for the two treatments (P = 0.045 for SED and P = 0.013 for CD).

The genes encoding to PepT1a and PepT1b (*slc15a1a* and *slc15a1b*, respectively) expressed only in the proximal two-thirds of the intestine, while the gene encoding to PepT2 (*slc15a2*) expressed mostly in the distal two-thirds of the intestine (Fig 2). The relative expression of these genes was affected by the NaCl level in the feed. Fish that were fed SED showed a significantly (P<0.05) higher expression of *slc15a1a* at the AI (Fig 2A), and significantly (P<0.05) higher expression of *slc15a1b* and *slc15a2* at the MI than the control fish (Fig 2B and 2C). The relative expression levels of these three genes were tested for linear correlation with the ADC of protein, for each diet and intestinal section. Significant negative correlations were found between the expression levels of *slc15a2* at the posterior intestine and the ADC of protein for the two treatments (P = 0.045, r = -0.95 for SED and P = 0.013, r = -0.95 for the CD) (Fig 3).

### Ion transporters

**Fig 4:**
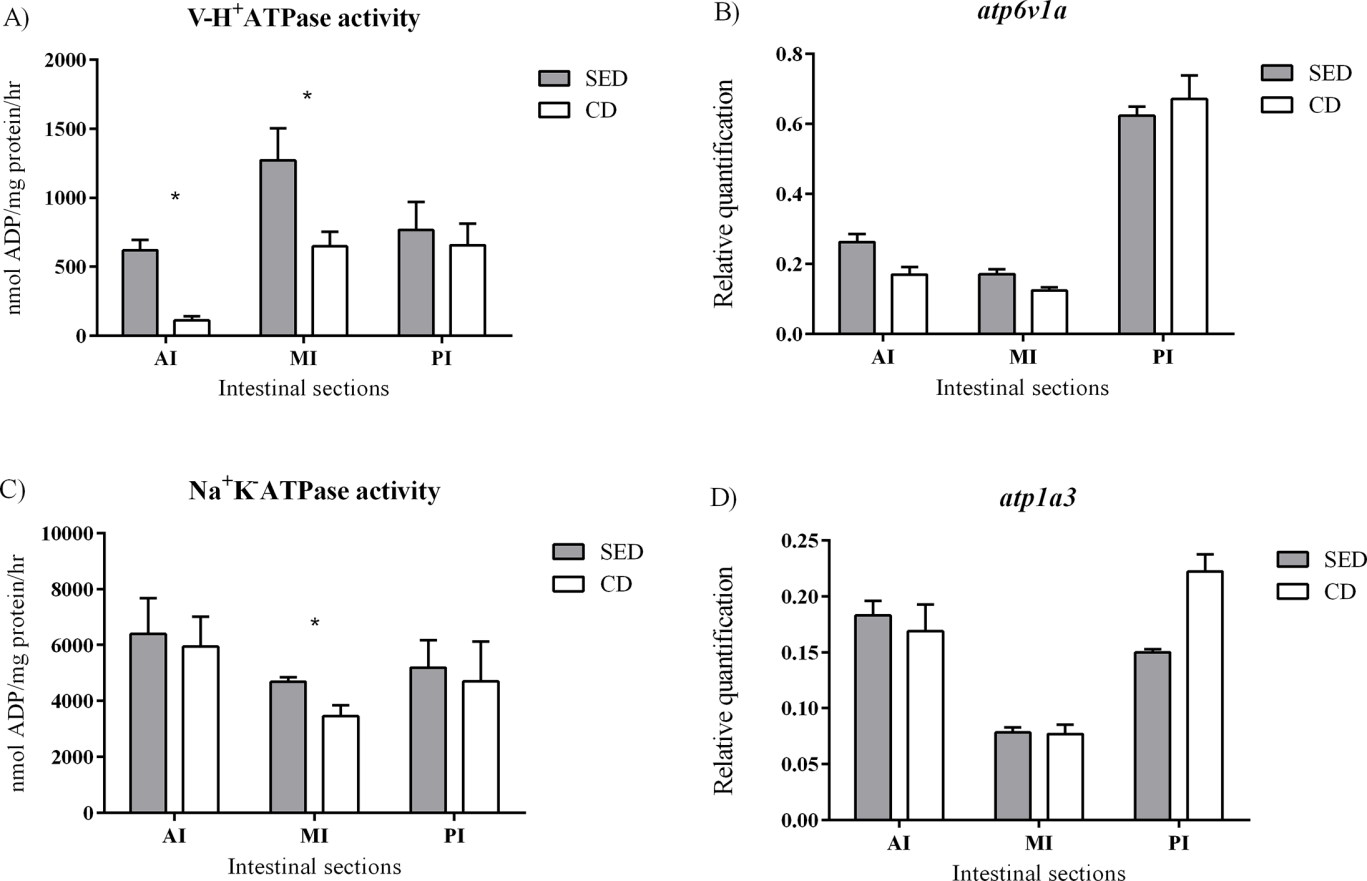
Activity assay of V-H^+^-ATPase (A) and Na^+^K^-^ATPase (C) and relative quantification of mRNA expression of *atp6v1a* (B) and *atp1a3* (D) at the anterior (AI), middle (MI) and posterior (PI) intestine between the treatments. The values for the activity assay are nmol ADP/ mg protein/hr. N = 6, asterisks indicate a significant difference between the treatments (P<0.05).

**Fig 5:**
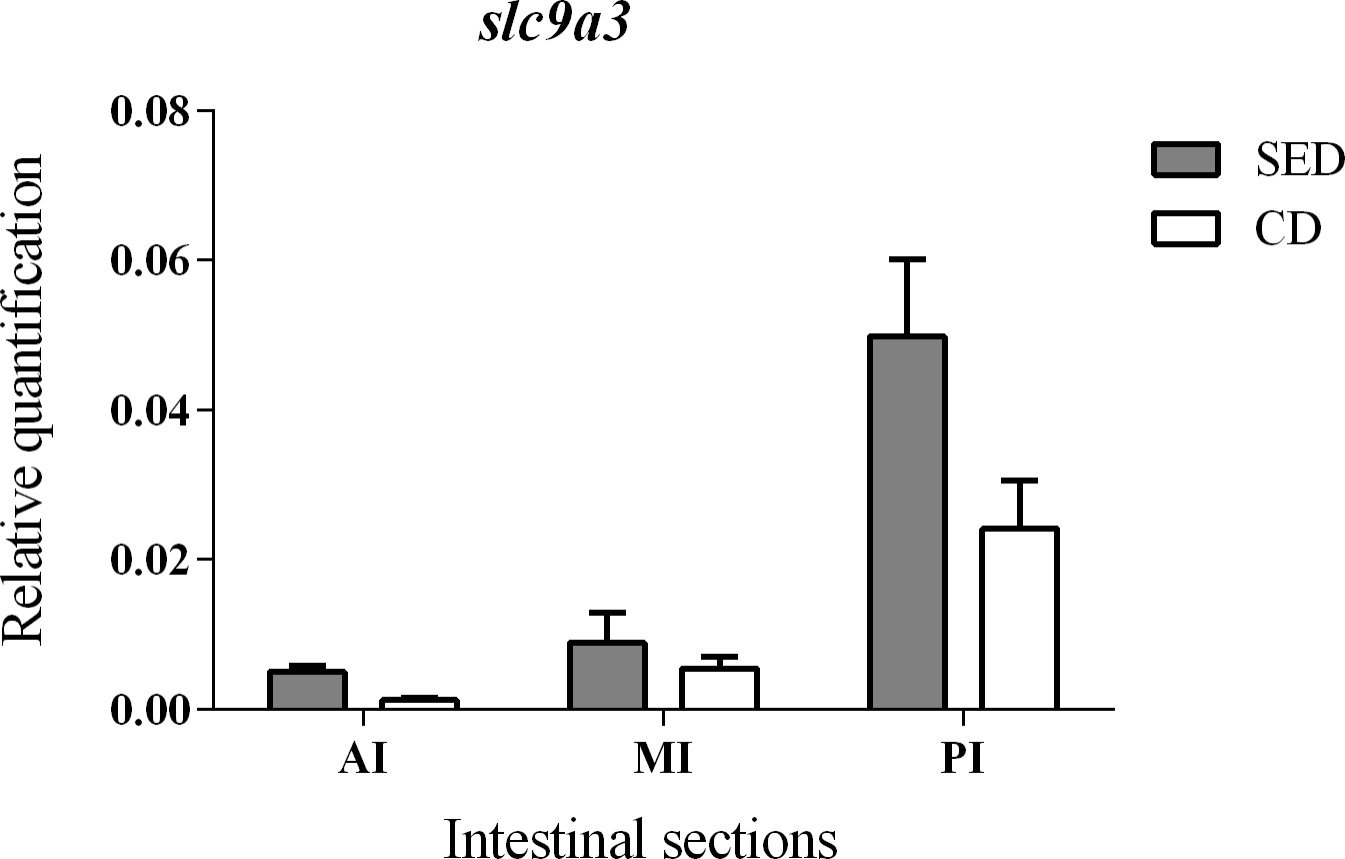
Relative quantification of mRNA expression of *slc9a3* (coding to NHE3) at the anterior (AI), middle (MI) and posterior (PI) intestine of fish fed the salt-enriched (SED) and the control (CD) diets. N = 6.

No significant differences in gene expression levels were found between the treatments for *atp6v1a*, *slc9a3* and *atp1a3* genes (coding for V-H^+^-ATPase, NHE3 and Na^+^/K^+^-ATPase, respectively) (Figs 4B, 4D and 5). However, fish that were fed with SED showed a significantly higher V-H^+^-ATPase activity at the AI and MI, relative to fish that were fed CD (Fig 4A) and a significantly higher Na^+^/K^+^-ATPase activity at the MI relative to fish that were fed CD (Fig 4C).

### Correlations in relative gene expression

When analyzing all the fish in the experiment, significant positive correlations were found between the relative expression of the genes coding for PepT1a and NHE3 in the AI (P = 0.02, r = 0.75), PepT1b and V-H^+^-ATPase in the MI (P = 0.007, r = 0.79), and PepT2 and V-H^+^-ATPase in the MI and PI (P = 0.01, r = 0.73; P = 0.02, r = 0.69, respectively). When analyzing fish from each dietary treatment separately, significant positive correlations were found between the relative expression of the genes coding for V-H^+^-ATPase and PepT2 in the MI of fish that were fed SED and PI of fish that were fed CD (P = 0.01, r = 0.96; P = 0.03, r = 0.86, respectively).

### Intestinal microbiome

**Fig 6:**
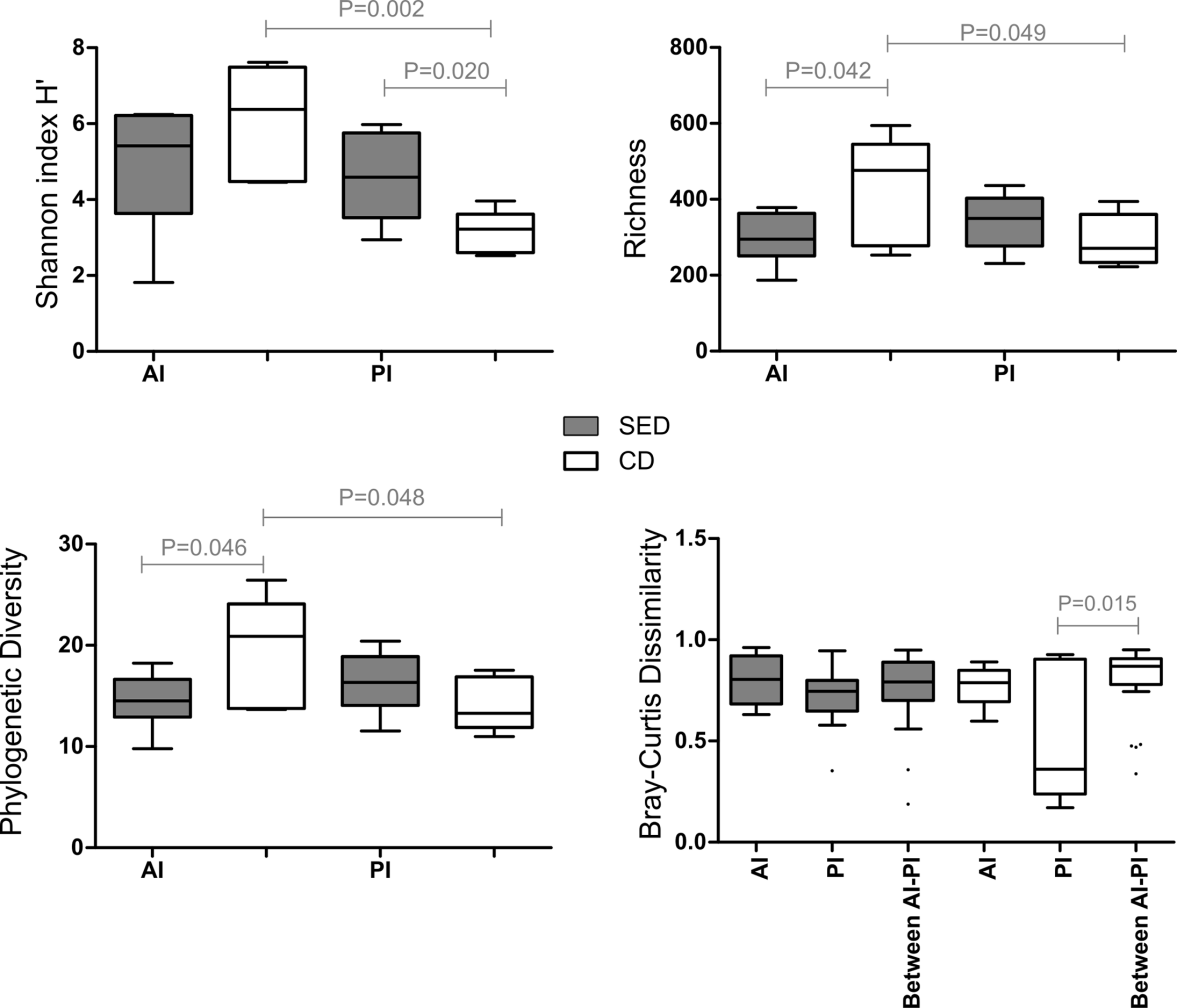
Microbial alpha-diversity measured by (A) Shannon H’, (B) richness, and (C) phylogenetic diversity indices in the anterior (AI) and posterior (PI) intestine between the treatments. (D) Microbial composition (beta diversity) measured by Bray Curtis index, comparing within and between parts dissimilarity of each group. Horizontal line in the box plots represents the median and whiskers indicate the lowest and highest point within 1.5 interquartile ranges of the lower or upper quartile, respectively. One-sided Student t-test was performed for the alpha diversity analysis (A-C), while for the Bray Curtis dissimilarity index, non-parametric P-values were calculated with 1000 Monte Carlo simulations and were Bonferroni-corrected.

The addition of salt to the diet had a significant effect on the PI microbial diversity, as Shannon H’ diversity was higher in the SED group compared to the CD group (P = 0.02; Fig 6A). In the AI, a slight but significant decrease was found in the microbial richness (P = 0.042; Fig 6B) and phylogenetic diversity (P = 0.046; Fig 6C). Looking within each group, significant differences between the two intestinal parts observed only in the CD group, in all indices (P = 0.002–0.049; Fig 6), but not in the SED group, indicating that in this group the parts tend to be more similar. This was also verified after comparing the dissimilarity within and between the two intestinal parts in each group, showing no difference in SED group, while in the CD group a high dissimilarity was found between AI and PI in relation to within each one (Fig 6D).

**Fig 7:**
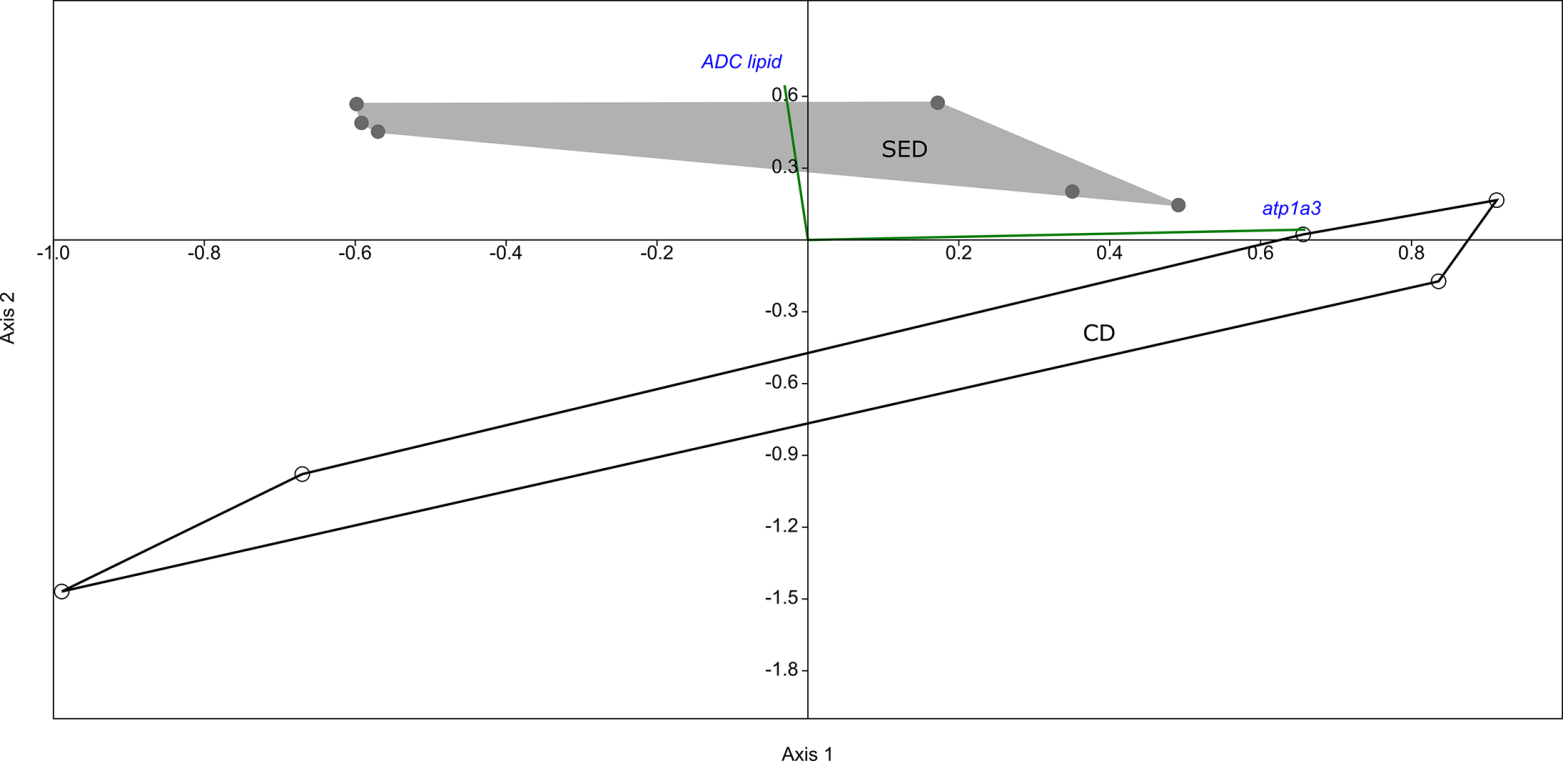
Canonical correspondence analysis (CCA) of the posterior intestine (PI) microbiome showing the two groups (SED and CD) forming two separate clusters, where each dot (either gray or white, respectively) is indicating an individual fish microbiome. The physiological parameters *ADC lipid* and *atp1a3* are plotted (green lines) as correlations with the PI microbiome of the treatments. The *ADC lipid* was positively correlating, and thus has a direction towards the SED group, while the *atp1a3* was negatively correlating with the SED group and thus positively with the CD group.

Correlation analysis using Spearman r showed that microbial diversity indices were associated with lipid digestibility and *atp1a3* (Na^+^/K^+^-ATPase) expression in the PI (Table 3). No significant correlations were found in the AI. Moreover, CCA revealed two separate clusters within the PI microbiome of individuals fed the two diets (Fig 7), explained by the lipid ADC and *atp1a3* expression.

**Table 3:** Spearman r correlation coefficient between the Shannon H’ and phylogenetic diversity indices in the posterior intestine (PI) and fish growth and physiological parameters.

	Shannon H’ diversity		Phylogenetic diversity	
	r	P-value	r	P-value
SGR	0.018	0.96	-0.05	0.89
ADC protein	0.44	0.18	0.46	0.15
**ADC lipid**	0.53	0.09	**0.61**	**0.04**
ADC ash	0.46	0.17	0.36	0.27
ADC dry matter	0.46	0.17	0.36	0.27
*slc15a2*	0.20	0.56	-0.32	0.34
*atp6v1a*	0.20	0.57	-0.50	0.11
***atp1a3***	**-0.80**	**0.003**	-0.14	0.68
*slc9a3*	-0.20	0.53	0.28	0.40
V-H^+^-ATPase	-0.49	0.98	-0.54	0.08
Na^+^/K^+^-ATPase	-0.05	0.87	0.00	0.12

Positive values indicate positive correlations and negative values indicate inverse correlations between Shannon H’ and phylogenetic diversity index in both groups (SED or CD), with each one of the physiological parameters. Significant values (P<0.05) are indicated in bold.

**Fig 8:**
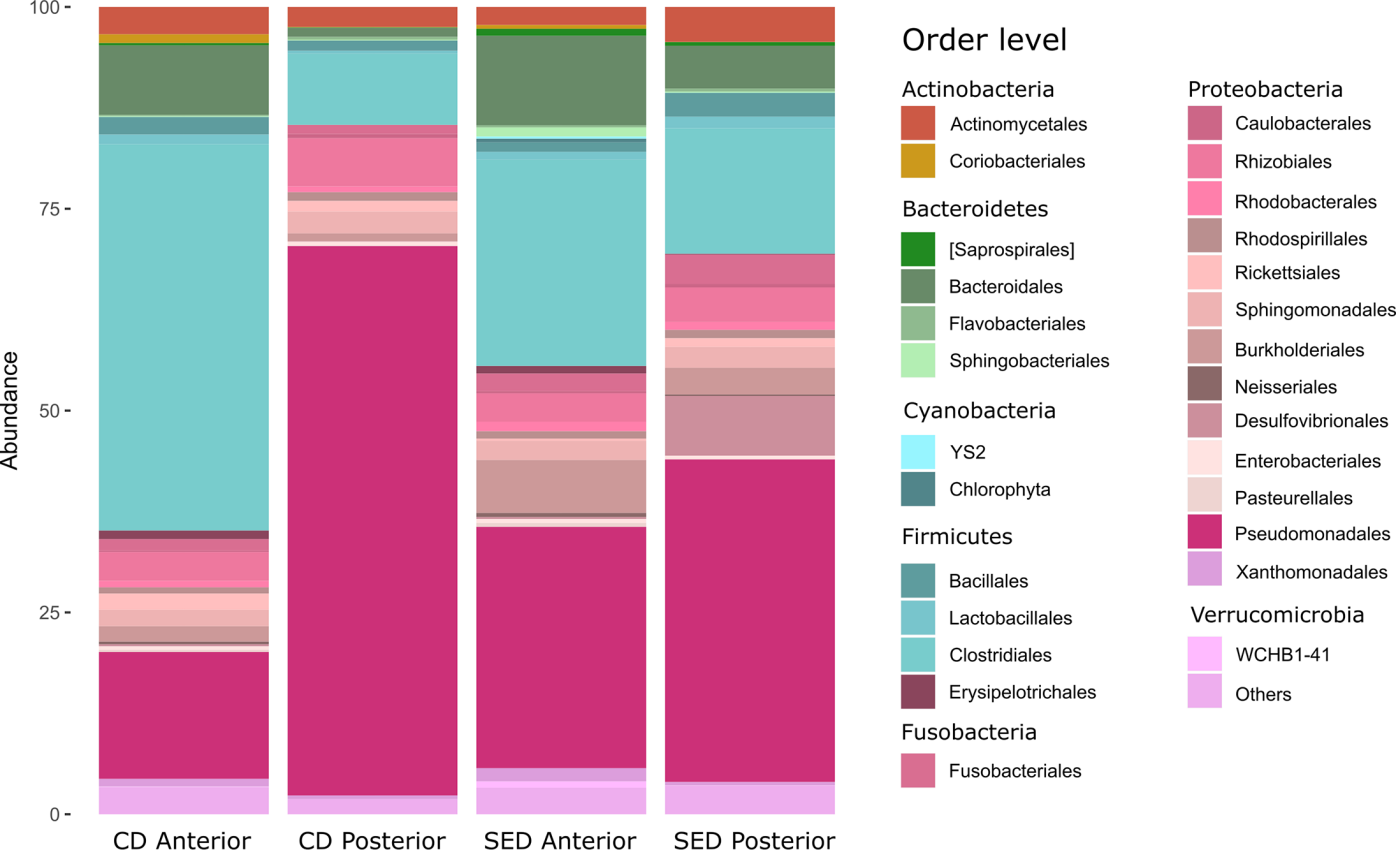
Relative abundance of the most abundant orders in the anterior and posterior intestine of the two groups. Different colors indicate the different taxa.

At the genus level, the microbial composition in the PI showed 57% dissimilarity between the two groups. Several genera were shown to contribute to this difference (Table 4; Fig 8). *Pseudomonas* contributed most of the dissimilarity between the samples (30%), being higher in the CD group, while genera of the family *Desulfovibrionaceae* contributed 6% of the total dissimilarity being enriched in the SED group. Significant enrichment in the *Micrococcus* taxa (1.8 vs 0.2% of the relative abundance) and genera of the family *Clostridiaceae* (1.48 vs 0.13% of the relative abundance) was found in the SED group compared to the CD (P = 0.04 and P = 0.04, respectively), contributing to 1.5 and 1.2% of the dissimilarity, respectively (Table 4). Moreover, dietary salt addition significantly increased the abundance of the genera *Succiniclasticum* (0.35 vs 0.01% of the relative abundance) and *Sediminibacterium* (0.23 vs 0% of the relative abundance) in the PI (Table 4).

**Table 4:** Similarity percentages (SIMPER) indicating the most abundant genera contributing to the discrimination between groups.

Genus	Average dissimilarity	Contribution %	Mean relative abundance SED	Mean relative abundance CD
*Pseudomonas*	17.26	30.3	0.344	0.546
*Enhydrobacter*	6.33	11.12	0.0307	0.116
Desulfovibrionaceae*	3.54	6.218	0.0708	6.75E-05
***Butyrivibrio***	3.36	5.895	0.0711	0.0601
*Cetobacterium*	1.16	2.789	0.0326	0.0071
*Prevotella*	1.26	2.22	0.0264	0.00208
***Clostridium***	1.17	2.052	0.0239	0.00459
*Micrococcus*	0.86	1.518	0.0188	0.002
Clostridiaceae*	0.69	1.215	0.0148	0.00136
*Sediminibacterium*	0.17	0.3028	0.00349	0.0001
***Succiniclasticum***	0.12	0.2078	0.00237	0

The average Bray Curtis dissimilarity was 57%, which is the overall Bray Curtis dissimilarity between samples of the two compared groups. Average dissimilarity defines the contribution of a genus to the average dissimilarity between groups. The contribution is the percentage of a genus from the dissimilarity between the groups. The mean relative abundance is the average relative abundance of a genus within a group. Stars indicate the family level, where taxonomic information at the genus level was not available. Genera belonging to the Firmicutes phylum are highlighted in bold.

## Discussion

In a previous study conducted with tilapia, we found that feeding fish with dietary salt supplementation improved feed utilization and growth [8]. The goal of the current study was to examine the effects of dietary salt supplementation on factors related to feed utilization, such as nutrients digestibility, intestinal transporters and the microflora composition.

Our results show that the addition of NaCl to the fish feed increases the digestibility. ADC values depend on the feed composition as well as the marker used and the fecal collection methods [27,37,38]. Wang et al. [39] reported that the ADC values in Nile tilapia that were fed a diet containing 35% protein (derived from fish, rapeseed and soy bean meal) were 75% and 87% for dry matter and protein, respectively, similar to the results obtained in the present study for the fish that were fed CD. Jimoh et al. [25] reported similar results, showing that ADC of Nile tilapia fed a diet containing 30% protein (derived from fish, jack bean and soy bean meal) was 86% and 91% for protein and lipid, respectively. Better digestibility of dry matter leads to lower feces secretion to the water, thus causing less environmental pollution. In fact, per one gram of body weight, fish that were fed CD excreted almost 10% more than the fish that were fed SED.

The improvement of ash digestibility in the SED group could be a direct result of the NaCl supplementation. Among the ingredients that form the ash component, there is a large proportion of NaCl in the SED, which dissolved into ions that are easily absorbed in the gut. In freshwater, which is a hypotonic environment, fish need to invest energy to actively insert ions through the gills and the digestive system against the concentration gradient [1]. The dietary supplementation of NaCl increases the content of ions in the intestinal lumen, reduces the gradient, and therefore, probably leads to an enhancement of ion absorption.

The supplementation of NaCl to the feed improved protein digestibility. A recent study that analyzed chime composition in rainbow trout (*Oncorhynchus mykiss*) also found an effect of dietary NaCl on protein absorption [13]. The mechanism behind this NaCl-dependent increase in protein utilization is not known. Research on Asian sea bass (*Lates calcarifer*) reported that the addition of salt to the feed enhanced the activity of the proteolytic enzyme Leucine aminopeptidase in the intestinal brush border of the fish [12]. Higher activity of proteolytic enzymes should result in a higher amount of substrate (FAA and di/tri-peptides as hydrolysis products) for the enterocyte transporters.

After proteolytic breakdown, the absorption of protein components into the enterocyte is carried out by different transporters. In this research, we focused on transporters that transfer di/tri-peptides to the enterocyte, PepT1 and PepT2, which are considered to have an important role in fish nutrition [23,40]. The mRNA levels of these transporters were affected by dietary NaCl content, with higher expression in the group fed SED for all three transporters. This phenomenon can be explained by the concentration of protons, a substrate for the PepTs activity. The intestinal cells are a source for luminal protons derived from cellular metabolism and excreted to the lumen through the apical V-H^+^-ATPase pump and the NHE3 antiporter. For these two proteins, the gene expression was not affected by the dietary treatments, however, the biochemical activity of V-H^+^-ATPase was significantly higher in the fish that were fed SED. This increased activity in the SED treatment should result in higher proton concentration at the apical membrane of the enterocyte, protons that are the driving force for small peptides absorption into the enterocytes. The strong correlations that were found between gene expression levels of V-H^+^-ATPase and PepTs, in fish receiving both diets, are further indication of the V-H^+^-ATPase role as the driving force behind the proton gradient that enable PepTs activity, as previously suggested by Con et al. [4]. The significant positive correlation that was found between the gene expression of NHE3 and PepT1a at the AI might be an indication that this proton transporter also has some role in PepT activity, as seen in mammalians [3,16,17].

Until recently, PepT2 was not known to be expressed in the intestine, however, Con et al. [4] localized this transporter in the distal intestinal sections of Mozambique tilapia. In the present study, we have seen similar spatial expression of the PepT genes along the intestine of the Nile tilapia. This pattern is probably due to their kinetic properties. PepT1 has high capacity and low affinity to the di/tri-peptides substrate, while PepT2 has low capacity and high affinity to the di/tri-peptides substrate [19,20]. This means that PepT1 is more effective at high substrate concentrations, conditions prevailing in the proximal intestinal section, and PepT2 is more effective at low substrate concentrations, prevailing in the distal intestinal section.

As PepTs transport short peptides into the enterocyte, we expected to have a positive correlation between mRNA expression of the PepT genes and the digestibility of the protein. Indeed, when comparing the fish receiving the two different diets, those consuming dietary NaCl supplementation had both higher PepTs expression levels and higher protein digestibility values (Figs 1 and 2). However, within each dietary treatment, in contrast to our expectation, we found that there is a negative correlation between the PepT2 expression and protein digestibility (Fig 3). One explanation for this phenomenon could be that in fish that are highly efficient in protein digestion, the majority of the di/tri-peptides were absorbed in the anterior and middle intestine, resulting in low concentration in the posterior intestine, which led to down-regulation of the PepT2 gene expression. Yet, under such conditions we would expect a positive correlation between PepT1 expression in the proximal intestinal sections and protein digestibility, such a correlation was not found. Other explanations as to why PepT gene expression does not correlate with protein digestibility might be differences in the intestinal lumen conditions that affect transporters efficiency rather than expression, or that protein digestibility relies more on FAA absorption rather than on di/tri-peptide.

The involvement of the gut microflora in the processes of digestion and absorption of the host is widely recognized, including in fish [41]. Factors such as diet composition are dominant in shaping gut microbial communities in both mammals and fish [41–43]. However, there are very limited studies examining the effect of dietary salt in the levels on intestinal microbial communities. A single work in the European sea bass (*Dicentrarchus labrax*) have shown that addition of salt to low fish meal diets made the pyloric caeca microbial communities more similar to the high fish meal diets [14]. Our results show that dietary salt supplementation increased the microbial diversity in the posterior intestine, while on the same time made the microbial composition across the gut to become more similar, unlike the control fed fish where the microbial communities clearly discriminate (higher diversity in the anterior and lower in the posterior intestine). Previous study in the Mozambique tilapia shown that there is a salinity-dependent localization shift of the intestinal transporters toward the distal sections of the intestinal tract when the fish were grown in seawater. A potential explanation could be due to the fish higher drinking rate in saltwater than in freshwater, resulting in rapid movement of nutrients down the intestine [4]. If this is the case in our study, then we would expect to have a higher level of nutrients in the posterior part of the intestine, which could possibly enrich the microbial communities in this compartment.

The increase of microbial diversity in the posterior intestine can also explain the positive correlation of the microbial diversity with lipids digestibility. It has been previously reported in zebrafish that the gut microbiota stimulates fatty acids uptake and impact host’s energy balance by enriching the Firmicutes phylum [44]. Although we did not find a statistically significant diet related enrichment of this phylum in the posterior intestine (in SED Firmicutes occupied 20% and in CD 10% of the overall relative abundance; Fig 8), we did observe a higher abundance of specific Firmicutes taxa. Moreover, we found a significant enrichment of *Micrococcus* genus. This genus has been reported as a probiotic in the Nile tilapia, preventing disease and enhancing growth when added in the feeds [45]. The exact mechanism of this improvement is not yet understood, but a potential change in the intestinal microbial balance could have accelerated food absorption.

In summary, the results of this study provide evidence that in Nile tilapia the addition of salt to the diet, increases the digestibility of protein, lipid, ash and dry matter. We have demonstrated that dietary NaCl content affects expression of PepT genes, although this is probably not the causative reason for the increased protein digestibility. Further research is needed in order to characterize the relationship between dietary ions and nutrient transporters in the context of feed efficiency, but also to better understand their connection with the gut microflora.
